# Household income and medical help-seeking for fertility problems among a representative population in Japan

**DOI:** 10.1186/s12978-021-01212-w

**Published:** 2021-08-03

**Authors:** Arisa Iba, Eri Maeda, Seung Chik Jwa, Ayako Yanagisawa-Sugita, Kazuki Saito, Akira Kuwahara, Hidekazu Saito, Yukihiro Terada, Osamu Ishihara, Yasuki Kobayashi

**Affiliations:** 1 Department of Public Health, Graduate School of Medicine, The University of Tokyo, 7‐3‐1 Hongo, Bunkyo‐ku, Tokyo, 113-0033 Japan; 2 Department of Environmental Health Science and Public Health, Akita University Graduate School of Medicine, 1-1-1 Hondo, Akita, Akita 010-8543 Japan; 3 Department of Obstetrics and Gynecology, Saitama Medical University, 38 Morohongo, Moroyama-machi, Iruma, Saitama 350-0495 Japan; 4 Department of Pediatrics, Perinatal, and Maternal Medicine (Ibaraki), Graduate School, Tokyo Medical and Dental University, 1-5-45 Yushima, Bunkyo-ku, Tokyo, 113-8510 Japan; 5 Department of Obstetrics and Gynecology, Graduate School of Biomedical Sciences, Tokushima University, 3-18-15 Kuramoto-cho, Tokushima, Tokushima 770-8503 Japan; 6 Umegaoka Women’s Clinic, 1-33-3 Umegaoka, Setagaya-ku, Tokyo, 154-0022 Japan; 7 Department of Obstetrics and Gynecology, Akita University Graduate School of Medicine, 1-1-1 Hondo, Akita, Akita 010-8543 Japan

**Keywords:** Infertility, Care-seeking, Healthcare disparities, Socioeconomic status, Japan

## Abstract

**Background:**

Fertility treatments help many infertile couples to have children. However, disparities exist in access to fertility tests and treatments. We investigated the association between household income and medical help-seeking for fertility in Japan.

**Methods:**

We conducted a cross-sectional study using nationally representative data from the National Fertility Survey 2015. Respondents were 6598 married women younger than 50 years old. The primary outcome was medical help-seeking for fertility among those who experienced fertility problems. Multiple logistic regression models were used to assess the association between household income and medical help-seeking, adjusting for age, length of marriage, educational level, employment status, number of children, childbearing desires, living with parents, and region of residence.

**Results:**

Among 2253 (34%) women who experienced fertility problems, 1154 (51%) sought medical help. The proportion of help-seekers increased linearly from 43% in the low-income group (< 4 million Japanese yen [JPY]) to 59% in the high-income group (≥ 8 million JPY) (*P* for trend < 0.001). Respondents with upper-middle (6–8 million JPY) or high household income were more likely to seek medical help, compared to those with low household income: adjusted odds ratio [aOR] 1.37 (95% confidence interval [CI]: 1.00–1.86) and aOR 1.78 (95% CI: 1.29–2.47), respectively.

**Conclusions:**

We found that higher household income was associated with a higher probability of seeking medical help among Japanese women who experienced fertility problem. Along with policy discussion about additional financial support, further studies from societal, cultural, or psychological views are required.

**Supplementary Information:**

The online version contains supplementary material available at 10.1186/s12978-021-01212-w.

## Plain Language Summary

Fertility treatments have helped millions of people to have a child. Although financial factors are known to play an important role in the decision to use fertility treatments, no previous studies have investigated how socioeconomic factors affect medical help-seeking for fertility in Japan. Therefore, we assessed the association between household income and medical help-seeking among couples with fertility problems in Japan, using nationally representative data from the National Fertility Survey 2015. Of the 6598 respondents (married women under 50 years old), one-third (2253) reported worrying about fertility problems, and half of those women (1154) sought medical help for fertility-related issues. The proportion of help-seekers was highest (59%) among those with high household income and lowest (43%) among those with low household income with a significant linear trend. Even after accounting for age, length of marriage, educational level, employment status, and other possibly related factors, those with higher household income were more likely to seek medical help for fertility tests and treatments. Japan provides various sources of financial support for fertility-related care, such as health insurance coverage for tests and early-stage treatments and partial subsidies for assisted reproductive technology treatments. However, the results indicate that further policy discussion about additional financial support and further studies focusing on barriers to care in Japan could help improve the situations for those with fertility problems.

**Supplementary Information:**

The online version contains supplementary material available at 10.1186/s12978-021-01212-w.

## Background

Infertility is a global public health issue [1, 2]. The need for and utilization of fertility treatments is increasing [3–6] as more people delay parenthood [7–9]. Fertility treatments have helped millions of people to have a child, but disparities in access to care persist. Various factors (e.g., financial, social, demographic, or psychological) contribute to these disparities [10, 11]. Financial accessibility plays a particularly important role in the decision to use fertility treatments [8, 12]. Among women who report infertility, those with higher income are more likely to seek a medical evaluation for infertility [13] and to use multiple and advanced treatments [12, 14, 15]. Even in Germany and Australia, where the public healthcare systems cover fertility treatments, patients living in high-income areas use more assisted reproductive technology (ART) cycles [16, 17]. Besides income, the cost is a critical factor in access to treatment; for example, reduction in out-of-pocket expenses with mandated health insurance coverage for fertility care is associated with increased help-seeking and utilization of fertility treatments in the United States [18, 19].

In Japan, where the total fertility rate is low (1.42 in 2018) and the mean parental age at first birth is high (30.7 and 32.8 years for women and men, respectively, in 2018) [20], 18.2% of married couples receive medical examinations or treatments for infertility, according to a national survey [21]. With Japan’s universal health insurance coverage [22], people can receive medical tests for fertility problem including hormonal test, hysterosalpingography, and early-phase fertility treatments including ovulation induction with timed intercourse using transvaginal ultrasonography with 30% copayments. On the other hand, intrauterine insemination and ART treatments are not covered by public health insurance. Alternatively, the government offers partial reimbursement of 300,000 Japanese Yen (JPY) (i.e., about 2,800 US dollars [USD]) per ART cycle for up to six cycles, for women younger than 43 [23], and the cost per fresh cycle in Japan is relatively low in the world [24], at approximately 400,000 JPY [25], or 3700 USD using the 2019 exchange rate of 1 USD = 109 JPY.

Japan provides adequate financial accessibility to fertility treatments, compared to most developed countries [26]. However, given the disparities observed in countries providing public funding for fertility treatments [16, 17], financial barriers to fertility care may still exist in Japan [27]. To date, no previous study has investigated the association between socioeconomic factors and medical help-seeking for fertility in Japan. Therefore, we aimed to explore the association between household income and medical help-seeking among couples with fertility problems in Japan, using nationally representative cross-sectional data from the National Fertility Survey.

## Methods

### Data source and study population

We used data from the Married Couples Survey of the 15th National Fertility Survey, conducted in June 2015. The National Fertility Survey was carried out by the National Institute of Population and Social Security Research under the Ministry of Health, Labour, and Welfare to collect nationally representative data on topics related to marriage, childbirth, and child-rearing in Japan since 1977 [21, 28]. The survey used a stratified, random cluster sampling method to select 900 enumeration districts from the 2010 Population Census [28]. Eligible candidates for the Married Couples Survey were married women younger than 50 years old who lived in these 900 districts, including foreign residents who spoke Japanese [28]. Eligible people received a self-administered questionnaire through home visits, and upon completion, returned it in a sealed envelope at a follow-up visit [21, 29]. Respondents provided information about their husbands and themselves. The number of valid responses was 6598 (87.8% collection rate) [21]. Data on primary sampling units and stratification were not available due to constraints on secondary data usage.

### Fertility status and medical help-seeking

Participants were asked whether they had ever worried about fertility problems. We categorized as subfertile those who reported current or previous subfertility by choosing the answer, “We (i.e., my husband and I) are currently worried about not being able to have a child” or “We were worried about not being able to have a child in the past.” We categorized as fertile those participants who chose the answer, “We have never worried about not being able to have a child.” Since the National Fertility Survey asked only about couples’ fertility concerns, not infertility by medical definition [2], we defined fertile and subfertile according to respondents’ perceptions of their relative fertility.

Subfertile participants were asked whether they had ever sought medical help for fertility problems. Those who chose the answer “We have never sought medical help” were categorized as non-help-seekers. Those who chose “We are currently undergoing testing or treatment” or “We have been tested or treated in the past” were categorized as help-seekers.

### Household income and other possible related factors

The participants reported their own and their spouse’s annual incomes from the previous year in increments of one million JPY (e.g., ≥ 2 million JPY and < 3 million JPY). We estimated annual household income by summing the midpoint value of each increment (e.g., 2.5 million JPY) for both participant and husband and then categorized income into four groups: low (< 4 million JPY), lower-middle (≥ 4 million JPY to < 6 million JPY), upper-middle (≥ 6 million JPY to < 8 million JPY), and high (≥ 8 million JPY).

We used the following sociodemographic variables as possible factors related to help-seeking: age (i.e., ≤ 29, 30–34, 35–39, ≥ 40), educational level (i.e., high school education or less, vocational or junior college education, university education or higher), employment status of participant and spouse at the time of the survey (i.e., full-time, part-time, self-employed, unemployed), length of marriage (i.e., ≤ 4 years, 5–9 years, 10–14 years, ≥ 15 years), number of existing children at the time of the survey (0, 1, 2, or ≥ 3), desire to have one or more children at the time of the survey (yes/no), living with their parents after marriage (yes/no), residential region (i.e., seven regions of Japan), and population size and density of the residential district (i.e., non-densely inhabited, less than 200,000 inhabitants, 200,000 to 1,000,000 inhabitants, more than 1,000,000 inhabitants) [30]. We gave an affirmative score to the desire to have additional children at the time of the survey if participants’ ideal number of children exceeded their reported number of children at that time. No questions were asked about respondents’ ethnicity, race or nationality, since the proportion of foreign residents in Japan is low (1.4% as of 2015) [31] and ethnicity questions are sensitive to some Japanese.

### Statistical analysis

We described the distributions of sociodemographic variables according to fertility status. Chi-square tests were used to compare the proportions of nominal variables (i.e., employment status, desire to have a child, living with parents, and region of residence), and a Wilcoxon-type test for trend was used to compare the proportions of ordinal variables (e.g., age and length of marriage) between fertile and subfertile groups.

The primary outcome was medical help-seeking among those who experienced fertility problems. Thus, we conducted the following analyses using the subfertile subgroup. We described the distribution of the variables according to help-seeking behavior and then used chi-square tests to compare the proportion of nominal variables and Wilcoxon-type tests for trend to compare the proportion of ordinal variables between help-seekers and non-help-seekers. Univariable logistic regression was used to explore the association of each variable with help-seeking.

We conducted multiple logistic regression analyses to assess the association between household income and medical help-seeking, controlling for the other covariates. We excluded the husband’s age from the multivariable model due to multicollinearity with the wife’s age. Although the proportion of missing values was small (< 6%) for all variables, 386 (17%) subfertile respondents had missing data for one or more investigated variable. Thus, in addition to the complete case analysis, we conducted multiple imputations for the multivariable regression analysis using the multivariate normal imputation method to account for missing data. We included all explanatory variables and the outcome variable [32] (1.2% missingness) in the imputation model, creating 20 multiply imputed data sets. Categorical variables were imputed using a set of binary indicators for each category, and unrounded values were used for the analysis [33].

We also conducted sensitivity analyses using data of respondents who reported current subfertility to analyze whether the association of household income and help-seeking at the time when they faced fertility problems is consistent. We omitted variables regarding husbands’ age, educational level, and employment status, living with parents, and residential regions to prevent overfitting. A two-sided *P* value of < 0.05 was used to define statistical significance. All analyses were performed using Stata version 14.2 (StataCorp., College Station, TX, USA).

### Ethical considerations

The study protocol was approved by the institutional review board of the Graduate School of Medicine at The University of Tokyo (approval no: 2019270NI, approved on January 23, 2020) and by the ethics committee of Akita University Graduate School of Medicine (approval no: 2300, approved on September 20, 2019). The Ministry of Health, Labor and Welfare approved the secondary use of data from the National Fertility Survey, and informed consent was not required because of anonymous data.

## Results

Of the 6598 valid respondents (i.e., wives), 341 (5%) and 1912 (29%) reported current and previous fertility problems, respectively. Thus, 2253 (34%) respondents were categorized as subfertile (Table 1). Wives and husbands of the subfertile group were significantly more educated and had longer marriages, higher household incomes, and fewer children at the time of the survey than those of the fertile group (all *P*s < 0.05). The proportion of those desiring one or more child at the time of the survey was significantly higher in the subfertile group (68%) than in the fertile group (42%, *P* < 0.001).

**Table 1:** Sociodemographic characteristics of participants according to fertility status and medical help-seeking

	Fertile (n = 3812)		Subfertile (n = 2253)		p^a^	Subfertile (aforementioned)				p^a^
						Non-help-seekers (n = 1071)		Help-seekers (n = 1154)		
	n	%	n	%		n	%	n	%	
Wife’s age, years										
≤ 29	310	8.1	135	6.0	0.80	87	8.1	47	4.1	< 0.001
30–34	516	14	339	15		183	17	151	13	
35–39	763	20	542	24		272	25	264	23	
≥ 40	2223	58	1237	55		529	49	692	60	
Husband’s age, years										
≤ 29	210	5.6	95	4.2	0.40	63	5.9	30	2.6	< 0.001
30–34	446	12	290	13		173	16	114	10	
35–39	621	16	447	20		227	21	215	19	
≥ 40	2504	66	1,404	63		599	56	787	69	
Length of the marriage, years										
≤ 4	546	15	415	19	< 0.001	248	24	161	14	< 0.001
5–9	703	19	557	25		275	26	277	25	
10–14	760	20	512	23		219	21	287	25	
≥ 15	1708	46	720	33		305	29	405	36	
Wife’s educational level										
High school education or less	1586	42	694	31	< 0.001	359	34	326	28	0.01
Vocational or junior college education	1473	39	985	44		452	42	520	45	
University education or higher	747	20	568	25		257	24	305	26	
Other	5	0.1	2	0.1		0	0.0	2	0.2	
Husband’s educational level										
High school education or less	1659	44	788	35	< 0.001	416	39	362	31	< 0.001
Vocational or junior college education	670	18	428	19		201	19	223	19	
University education or higher	1451	38	1025	46		446	42	565	49	
Other	9	0.2	2	0.1		0	0.0	2	0.2	
Wife’s employment status										
Full-time worker	849	23	577	26	< 0.001	283	27	286	25	0.78
Part-time worker	1543	41	772	35		361	34	400	35	
Self-employed	222	5.9	119	5.4		58	5.5	61	5.3	
Unemployed	1125	30	752	34		350	33	394	35	
Husband’s employment status										
Full-time worker	2829	79	1,739	81	0.20	815	81	905	82	0.90
Part-time worker	241	6.7	137	6.4		69	6.9	67	6.0	
Self-employed	448	13	235	11		110	11	122	11	
Unemployed	57	1.6	27	1.3		13	1.3	14	1.3	
Household income^b^										
Low	659	18	330	16	0.001	186	19	139	13	< 0.001
Lower-middle	945	26	527	25		263	26	258	24	
Upper-middle	844	23	519	24		249	25	268	24	
High	1145	32	743	35		302	30	429	39	
Number of existing children										
0	404	11	507	23	< 0.001	241	23	259	23	0.06
1	832	22	738	33		326	31	404	35	
2	1812	48	785	35		381	36	394	34	
≥ 3	740	20	211	9.4		118	11	92	8.0	
Living with parents										
Yes	1076	29	562	25	0.004	270	26	283	25	0.80
No	2639	71	1645	75		785	74	844	75	
Desire to have a child										
Yes	1552	42	1503	68	< 0.001	709	68	780	69	0.51
No	2170	58	705	32		341	32	353	31	
Residential region										
Hokkaido	129	3.4	60	2.7	0.27	31	2.9	27	2.3	0.44
Tohoku	237	6.2	148	6.6		79	7.4	68	5.9	
Kanto	1262	33	776	34		375	35	393	34	
Chubu	794	21	480	21		231	22	244	21	
Kinki	625	16	354	16		154	14	195	17	
Chugoku/Shikoku	351	9.2	223	10		99	9.2	121	10	
Kyushu/Okinawa	414	11	212	9.4		102	10	106	9.2	
Population size and density										
Non-densely inhabited district	1089	29	591	26	0.06	298	28	287	25	0.01
< 200,000 inhabitants	909	24	555	25		286	27	260	23	
200,000 to 1,000,000 inhabitants	1089	29	643	29		279	26	358	31	
> 1,000,000 inhabitants	725	19	464	21		208	19	249	22	

*JPY* Japanese Yen

^a^Chi-squared test for nominal variables and Wilcoxon-type test for trend for ordinal variables

^b^Categorized into four groups: low (< 4 million JPY), lower-middle (≥ 4 million JPY to < 6 million JPY), upper-middle (≥ 6 million JPY to < 8 million JPY), and high (≥ 8 million JPY)

**Fig. 1:**
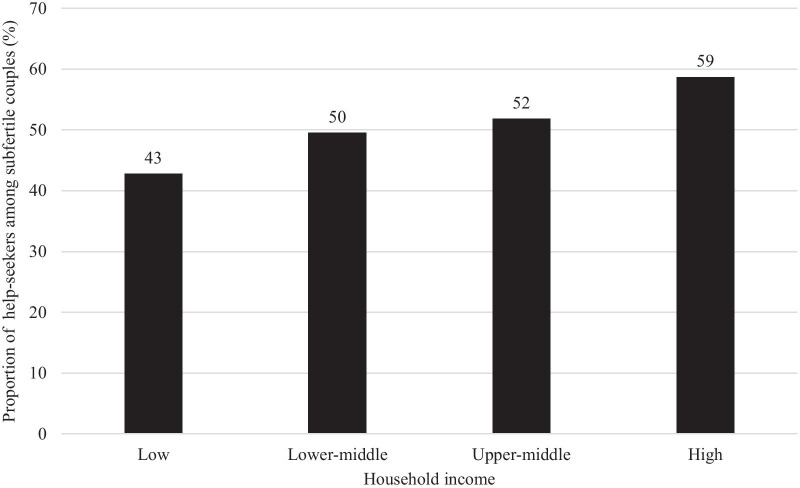
Proportions of medical help-seekers for fertility problems among subfertile couples in each income group

Among subfertile respondents, 1154 (51%) sought medical help for fertility problems (Table 1). Compared to non-help-seekers, medical help-seekers were significantly older and more educated, with longer marriages and higher household incomes, and they lived in more densely-inhabited districts (all *P*s < 0.05). The proportions of medical help-seekers in each income group were 43% in the low-, 50% in the lower-middle-, 52% in the upper-middle-, and 59% in the high-income groups with significant linear trend (*P* for trend < 0.001), respectively (Fig. 1). The proportions of those desiring a child at the time of the survey were similar between medical help-seekers and non-help-seekers.

Table 2 shows the results of the univariable and multivariable logistic regression analyses for medical help-seeking. In the univariable analyses, those with upper-middle or high household income were more likely to seek medical help for fertility problems, compared to those with low household income among subfertile respondents: odds ratio (OR) 1.44 (95% confidence interval [CI] 1.09–1.90) and OR 1.90 (95% CI 1.46–2.48), respectively. Also, older ages of wives and husbands, longer marriages, and higher education levels of wives and husbands were significantly associated with help-seeking for fertility problems.

**Table 2:** Logistic regression analyses for factors associated with medical help-seeking

	Univariable (n = 2225)			Multivariable (complete case analysis) (n = 1867)			Multivariable (multiple imputation) (n = 2253)		
	Odds ratio	95% CI	p	Odds ratio	95% CI	p	Odds ratio	95% CI	p
Household income^a^									
Low	Ref			Ref			Ref		
Lower-middle	1.31	0.99–1.73	0.06	1.30	0.92–1.83	0.14	1.28	0.95–1.73	0.10
Upper-middle	1.44	1.09–1.90	0.01	1.44	1.01–2.06	0.04	1.37	1.00–1.86	0.05
High	1.90	1.46–2.48	< 0.001	1.92	1.32–2.78	0.001	1.78	1.29–2.47	< 0.001
Wife’s age, years									
≤ 29	Ref			Ref			Ref		
30–34	1.53	1.01–2.31	0.05	1.15	0.71–1.86	0.58	1.15	0.74–1.79	0.52
35–39	1.80	1.21–2.66	0.003	1.14	0.71–1.86	0.59	1.16	0.74–1.79	0.52
≥ 40	2.42	1.67–3.51	< 0.001	1.22	0.74–2.01	0.45	1.28	0.81–2.02	0.29
Husband’s age, years									
≤ 29	Ref			–	–	–	–	–	–
30–34	1.38	0.84–2.27	0.20	–	–	–	–	–	–
35–39	1.99	1.24–3.19	0.004	–	–	–	–	–	–
≥ 40	2.76	1.76–4.32	< 0.001	–	–	–	–	–	–
Length of the marriage, years									
≤ 4	Ref			Ref			Ref		
5–9	1.55	1.20–2.01	0.001	1.85	1.34–2.55	< 0.001	1.74	1.30–2.33	< 0.001
10–14	2.02	1.55–2.63	< 0.001	2.33	1.61–3.38	< 0.001	2.30	1.63–3.23	< 0.001
≥ 15	2.05	1.60–2.62	< 0.001	2.42	1.61–3.64	< 0.001	2.47	1.70–3.59	< 0.001
Wife’s educational level									
High school education or less	Ref			Ref			Ref		
Vocational or junior college education	1.27	1.04–1.54	0.02	1.13	0.89–1.43	0.32	1.11	0.90–1.38	0.32
University education or higher	1.31	1.05–1.63	0.02	1.11	0.83–1.48	0.48	1.16	0.89–1.51	0.27
Husband’s educational level									
High school education or less	Ref			Ref			Ref		
Vocational or junior college education	1.27	1.01–1.62	0.05	1.28	0.98–1.69	0.07	1.25	0.97–1.61	0.08
University education or higher	1.46	1.21–1.76	< 0.001	1.22	0.96–1.56	0.10	1.27	1.02–1.59	0.03
Wife’s employment status									
Full-time worker	Ref			Ref			Ref		
Part-time worker	1.10	0.88–1.36	0.41	1.28	0.98–1.69	0.07	1.23	0.96–1.59	0.10
Self-employed	1.04	0.70–1.55	0.84	1.14	0.71–1.83	0.60	1.03	0.66–1.60	0.89
Unemployed	1.11	0.90–1.39	0.33	1.48	1.12–1.95	0.01	1.39	1.08–1.79	0.01
Husband’s employment status									
Full-time worker	Ref			Ref			Ref		
Part-time worker	0.87	0.62–1.24	0.45	0.79	0.53–1.18	0.24	0.85	0.58–1.25	0.41
Self-employed	1.00	0.76–1.31	0.99	0.98	0.70–1.38	0.92	1.10	0.80–1.50	0.55
Unemployed	0.97	0.45–2.08	0.94	1.11	0.47–2.63	0.82	1.33	0.59–3.00	0.49
Number of existing children									
0	Ref			Ref			Ref		
1	1.15	0.92–1.45	0.22	1.02	0.78–1.32	0.90	0.96	0.75–1.22	0.73
2	0.96	0.77–1.20	0.74	0.62	0.46–0.84	0.002	0.62	0.47–0.82	0.001
≥ 3	0.73	0.52–1.00	0.05	0.48	0.31–0.74	0.001	0.46	0.31–0.67	< 0.001
Living with parents									
No	Ref			Ref			Ref		
Yes	0.97	0.80–1.18	0.80	1.13	0.90–1.42	0.30	1.11	0.90–1.37	0.32
Desire to have a child									
No	Ref			Ref			Ref		
Yes	1.06	0.89–1.27	0.51	1.01	0.78–1.31	0.92	0.98	0.78–1.23	0.85
Residential region									
Hokkaido	Ref			Ref			Ref		
Tohoku	0.99	0.54–1.82	0.97	0.70	0.34–1.43	0.32	0.83	0.45–1.56	0.57
Kanto	1.20	0.70–2.05	0.50	0.69	0.36–1.32	0.26	0.78	0.45–1.36	0.38
Chubu	1.21	0.70–2.09	0.49	0.82	0.43–1.58	0.55	0.88	0.50–1.54	0.65
Kinki	1.45	0.83–2.54	0.19	0.92	0.47–1.78	0.80	1.02	0.58–1.81	0.95
Chugoku/Shikoku	1.40	0.79–2.51	0.25	0.94	0.47–1.87	0.86	1.12	0.62–2.03	0.72
Kyushu/Okinawa	1.19	0.67–2.14	0.55	0.88	0.44–1.76	0.72	1.05	0.58–1.91	0.87
Population size and density									
Non-densely inhabited district	Ref			Ref			Ref		
< 200,000 inhabitants	0.94	0.75–1.19	0.63	0.93	0.70–1.22	0.58	0.95	0.74–1.22	0.68
200,000 to 1,000,000 inhabitants	1.33	1.06–1.67	0.01	1.18	0.91–1.54	0.21	1.18	0.93–1.50	0.18
> 1,000,000 inhabitants	1.24	0.97–1.59	0.08	1.07	0.79–1.44	0.68	1.09	0.83–1.43	0.56

*CI* confidence interval, *JPY* Japanese Yen, *ref* reference

^a^Categorized into four groups: low (< 4 million JPY), lower-middle (≥ 4 million JPY to < 6 million JPY), upper-middle (≥ 6 million JPY to < 8 million JPY), and high (≥ 8 million JPY)

In the multivariable analysis using multiple imputations, participants with upper-middle or high household income were more likely to seek help for fertility problems, compared to participants with low household income, after adjusting for the covariates: adjusted OR (aOR) 1.37 (95% CI 1.00–1.86) and aOR 1.78 (95% CI 1.29–2.47), respectively. Longer marriages, husbands having university education, and unemployment of wives were positively associated with help-seeking. Participants who had two or more children were less likely to seek medical help for fertility problems than those who had no child at the time of the survey. A complete case analysis showed similar results to that of the multiple imputation methods.

Additional file 1: Table S1 shows distribution of sociodemographic factors among those who reported current subfertility. Multiple logistic regression analyses showed positive associations between household income and help-seeking among those with current subfertility, although the results were not statistically significant (Additional file 1: Table S2): aORs for upper-middle and high household incomes were 1.47 (95% CI 0.68–3.16) and 1.58 (95% CI 0.72–3.46), respectively.

## Discussion

Using a nationally representative survey, we investigated the association between household income and medical help-seeking among couples who experienced fertility problems. Approximately one third of participants reported fertility problems, for which half sought medical help. Couples with higher household income were more likely to seek medical help than those with lower household income. This study is the first to present the association between socioeconomic factors and help-seeking for fertility in Japan.

Among couples with fertility problems, 51% sought medical help. The proportion of help-seekers was similar or slightly lower than the international estimate of 56% reported by a previous systematic review [1] and by recent studies showing 57% in the United Kingdom [11] and 55% in China [34]. Among presumably health-conscious participants of the Nurses’ Health Study II in the United States, the proportion of those receiving medical evaluations for infertility was 65%, varying from 59% in the low-income group to 69% in the high-income group [13]. The National Fertility Survey in the current study asked participants to report their fertility concerns but not the experience of infertility, defined as “failing to achieve pregnancy after at least 12 months of unprotected regular sexual intercourse” [2]. Thus, the present proportion of help-seekers would not be comparable to previous works. Nonetheless, it remains a concern that a substantial proportion of couples did not seek medical help for their fertility problems.

The proportion of help-seekers linearly increased with household income, from 43% in the low-income group to 59% in the high-income group. The positive association between household income and medical help-seeking was consistent with previous studies conducted in other countries [12, 13, 35], even though Japan provides public health insurance coverage for fertility tests and early-phase treatments, as well as partial subsidies for ART treatments. A possible reason for this finding is that out-of-pocket payment remains expensive, relative to disposable income. For example, hysterosalpingography as a diagnostic testing for tubal factor infertility usually requires approximately 10,000 JPY (i.e., 90 USD) of copayment after health insurance coverage. Such out-of-pocket payments, as well as opportunity costs lost through medical consultations, might affect help-seeking, especially among lower-income groups. Some local governments provide subsidies for fertility tests and early-phase fertility treatments such as ovulation induction (e.g., up to 50,000 JPY in Tokyo Prefecture [36]), and some add local subsidies for ART treatments (e.g., additional 300,000 JPY per fiscal year [37] or additional three ART cycles [38]) to the national subsidy. As a future research direction, it would be necessary to evaluate the effect of such additional local subsidies on the improvements of medical help-seeking behavior among low-income group. Concerns about job security, housing security, and the cost of childcare and education may impose additional, indirect impacts on planned fertility [9]. These additional pressures are likely to compound the direct effects of income on access to fertility care, especially among those with lower household incomes.

We showed positive associations between husbands’ educational level and medical help-seeking, although wives’ education was not significant in the multivariable model (Table 2). Higher education is known to be associated with help-seeking behavior [11, 39]. Several explanations have been suggested from a non-economic perspective. Fertility knowledge [40] and awareness of fertility problems [41] may promote intentions to improve fertility [42] and increase access to health care [12]. Whereas help-seekers had positive treatment beliefs, such as perceived high success rates or ease of obtaining help, non-help-seekers may have more fear of discovering a problem and of being labeled infertile, as well as perceived high treatment costs. A recent local-government survey conducted in Japan revealed men’s fertility awareness to be lower than that of their wives, and some couples failed to pursue fertility care due to the husbands’ indifference [43]. A previous interventional study also found that fertility education increased new medical consultations for fertility, especially when the educational subjects were married men [44]. Thus, interventions for increasing fertility awareness, especially among men, could help more people receive treatment earlier.

Gender inequality in the home provides another potential explanation for the lack of association between wives’ education and help-seeking behavior. Domestic gender inequality may also explain the significant association between husbands’ education and help seeking. Traditional gender roles are still entrenched in Japanese societies, as manifested by the unequal division of domestic work [45]. Greater gender equality is positively correlated with ART utilization in Europe [47]. It is therefore possible that delayed help seeking and lack of cooperation by husbands are caused by gender inequality within couples as well as gender disparities in fertility literacy.

Unemployment of wives was associated with 1.5 times the odds of medical help-seeking, compared to full-time employment. Women with paid work were generally less likely to seek medical help for fertility [46]. As more and more couples in developed countries choose to remain childless [47], those experiencing less desire for children will be unlikely to seek medical help. However, we should note that women continue to experience difficulties balancing work with fertility plans. Once people sought medical help, they often discontinued fertility treatment due to the difficulty in integrating therapeutic programs with their work [48]. In Japan, 8% of working women quit their job due to fertility treatments [49]. Although our analyses were based on a cross-sectional survey and employment status was obtained at the time of the survey, employment would negatively affect seeking medical advice and receiving treatments. The low share of women in senior roles in Japan [50] suggests that it may be difficult for women to get permission to take time off work for infertility treatments, and then to return to their career track after pregnancy and childbirth. Furthermore, the wide gender gap in political empowerment [50] has delayed the development of legislation to correct these workplace disadvantages. As more women join the labor force, policies to increase public understanding of infertility and help employees balance work and fertility treatment are necessary.

To our knowledge, this is the first study to identify socioeconomic gaps in help-seeking for fertility problems in Japan. In the face of severe low fertility and increased infertility, the government has revised the public subsidy system for ART treatments many times [51] and recently decided to modify health insurance coverage for ART treatment starting in 2022 to reduce the financial burden for infertile patients [52]. With this coverage, more people who seek help will be able to receive advanced treatments. However, this policy change does not address those patients in Japan who are unlikely to seek help in the first place. Our study recommends future research and discussion to support people with fertility problems, including non-help-seekers.

Several limitations of this study should be acknowledged. First, the National Fertility Survey was a cross-sectional analysis, and thus we could obtain socioeconomic information at the time of the survey only. However, we confirmed similar associations between income and help-seeking behavior even among those with current fertility problems (Additional file 1: Table S2). Second, we could not fully account for the motivation to have a child, although desire to have a child was included in our multivariable models. As intentional childlessness is now widely accepted in developed countries [47], attitudes toward childbearing should be assessed in future research.

Third, the Married Couples Survey of the 15th National Fertility Survey evaluated legally married couples only. We were therefore unable to investigate attitudes to fertility across the full spectrum of the Japanese population. However, we believe our results were representative of national birth trends, because 98% of births in Japan are registered to legally married heterosexual couples [53]. A national survey on attitudes to fertility and children among non-heteronormative individuals and couples will be needed in the future.

Fourth, we performed the analyses without accounting for cluster sampling or stratification because data on stratification and sampling units were not available. Although this did not affect the point estimates [54, 55], the standard errors of the estimated odds ratio might have been affected: the lack of accounting for clustering could have led to an overestimate of the standard error, and the omission of stratification data could have led to an underestimate of the standard error [55]. Thus, confidence intervals in our analyses should be interpreted with caution. Finally, although the response rate to the National Fertility Survey was high (87.8%), non-respondents may have introduced selection bias into our findings. However, the distribution of age, employment status and educational level across the respondents were almost equivalent to that of the national population [31, 56].

## Conclusions

This study assessed the association between household income and the seeking of medical help for fertility problems using nationally representative survey data. Although Japan provides public funding for fertility treatments, we found that higher household income was associated with a higher probability of help-seeking among couples with fertility problems. Further in-depth studies to investigate related factors (e.g., financial, societal, cultural, and psychological) would be needed to inform future policy-making and improve the situations for those suffering from fertility problems.

## Supplementary Information

**Additional file 1. Table S1**. Sociodemographic characteristics of participants who had reported current fertility problems according to the status of medical help-seeking.** Table S2**. Logistic regression analysis for factors associated with medical help-seeking among participants who had reported current fertility problems.

## Data Availability

The datasets generated or analyzed during the current study are not publicly available due to restrictions on data-sharing for the National Fertility Survey of Japan, managed by the National Institute of Population and Social Security Research, under the Japanese Ministry of Health, Labour and Welfare.

## Abbreviations

ART: Assisted reproductive technology
JPY: Japanese Yen
USD: US dollars
OR: Odds ratio
CI: Confidence interval
aOR: Adjusted OR
