# Supplementary material for: Household income and medical help-seeking for fertility problems among a representative population in Japan
Source: Reprod Health. 2021 Aug 3;18:165. doi: 10.1186/s12978-021-01212-w (PMC8336394; doi:10.1186/s12978-021-01212-w)
Supplement: Supplementary file 1 — Additional file 1. Table S1. Sociodemographic characteristics of participants who had reported current fertility problems according to the status of medical help-seeking. Table S2. Logistic regression analysis for factors associated with medical help-seeking among participants who had reported current fertility problems. [file 12978_2021_1212_MOESM1_ESM.docx]

Table S1. Sociodemographic characteristics of participants who had reported current fertility problems according to the status of medical help-seeking.

|  |  | Non-help-seekers | |  | Help-seekers | |  |  |
| --- | --- | --- | --- | --- | --- | --- | --- | --- |
|  |  | (n=172) | |  | (n=169) | |  |  |
|  |  | n | % |  | n | % |  | p^a^ |
| Wife's age, years | |  |  |  |  |  |  | 0.08 |
|  | ≤29 | 26 | 15 |  | 15 | 8.9 |  |  |
|  | 30–34 | 45 | 26 |  | 45 | 27 |  |  |
|  | 35–39 | 62 | 36 |  | 60 | 36 |  |  |
|  | ≥40 | 39 | 23 |  | 49 | 29 |  |  |
| Husband's age, years | |  |  |  |  |  |  | 0.13 |
|  | ≤29 | 18 | 10 |  | 13 | 7.7 |  |  |
|  | 30–34 | 42 | 24 |  | 32 | 19 |  |  |
|  | 35–39 | 47 | 27 |  | 52 | 31 |  |  |
|  | ≥40 | 64 | 37 |  | 72 | 43 |  |  |
| Length of the marriage, years | |  |  |  |  |  |  | 0.23 |
|  | ≤4 | 81 | 47 |  | 61 | 36 |  |  |
|  | 5–9 | 55 | 32 |  | 70 | 41 |  |  |
|  | 10–14 | 24 | 14 |  | 26 | 15 |  |  |
|  | ≥15 | 10 | 5.8 |  | 9 | 5.3 |  |  |
| Wife's academic background | |  |  |  |  |  |  | 0.22 |
|  | Highschool education or less | 53 | 31 |  | 40 | 24 |  |  |
|  | Vocational or junior college education | 70 | 41 |  | 76 | 45 |  |  |
|  | University education or higher | 49 | 28 |  | 53 | 31 |  |  |
|  | Other | 0 | 0 |  | 0 | 0 |  |  |
| Husband's academic background | |  |  |  |  |  |  | 0.06 |
|  | Highschool education or less | 72 | 42 |  | 51 | 30 |  |  |
|  | Vocational or junior college education | 29 | 17 |  | 36 | 21 |  |  |
|  | University education or higher | 71 | 41 |  | 81 | 48 |  |  |
|  | Other | 9 | 5.2 |  | 2 | 1.2 |  |  |
| Wife's employment status | |  |  |  |  |  |  | 0.21 |
|  | Full-time worker | 56 | 33 |  | 54 | 32 |  |  |
|  | Part-time worker | 48 | 28 |  | 64 | 38 |  |  |
|  | Self-employed | 7 | 4.1 |  | 5 | 3.0 |  |  |
|  | Unemployed | 60 | 35 |  | 46 | 27 |  |  |
| Husband's employment status | |  |  |  |  |  |  | 0.30 |
|  | Full-time worker | 123 | 72 |  | 129 | 76 |  |  |
|  | Part-time worker | 15 | 8.7 |  | 16 | 9.5 |  |  |
|  | Self-employed | 23 | 13 |  | 12 | 7.1 |  |  |
|  | Unemployed | 4 | 2.3 |  | 3 | 1.8 |  |  |
| Household income^b^ | |  |  |  |  |  |  | 0.08 |
|  | Low | 29 | 17 |  | 22 | 13 |  |  |
|  | Lower-middle | 55 | 32 |  | 48 | 28 |  |  |
|  | Upper-middle | 33 | 19 |  | 41 | 24 |  |  |
|  | High | 45 | 26 |  | 56 | 33 |  |  |
| Number of existing children | |  |  |  |  |  |  | 0.09 |
|  | 0 | 110 | 64 |  | 117 | 69 |  |  |
|  | 1 | 49 | 28 |  | 50 | 30 |  |  |
|  | 2 | 10 | 5.8 |  | 2 | 1.2 |  |  |
|  | ≥3 | 1 | 0.6 |  | 0 | 0 |  |  |
| Living with their parents | |  |  |  |  |  |  | 0.36 |
|  | Yes | 129 | 75 |  | 118 | 70 |  |  |
|  | No | 40 | 23 |  | 46 | 27 |  |  |
| Residential region | |  |  |  |  |  |  | 0.84 |
|  | Hokkaido | 6 | 3.5 |  | 8 | 4.7 |  |  |
|  | Tohoku | 15 | 8.7 |  | 10 | 5.9 |  |  |
|  | Kanto | 57 | 33 |  | 55 | 33 |  |  |
|  | Chubu | 39 | 23 |  | 34 | 20 |  |  |
|  | Kinki | 28 | 16 |  | 28 | 17 |  |  |
|  | Chugoku/Shikoku | 17 | 9.9 |  | 19 | 11 |  |  |
|  | Kyushu/Okinawa | 10 | 5.8 |  | 15 | 8.9 |  |  |
| Population size and density | |  |  |  |  |  |  | 0.19 |
|  | Non-densely inhabited district | 44 | 26 |  | 35 | 21 |  |  |
|  | <200,000 inhabitants | 46 | 27 |  | 47 | 28 |  |  |
|  | 200,000 to 1,000,000 inhabitants | 48 | 28 |  | 42 | 25 |  |  |
|  | ≥1,000,000 inhabitants | 34 | 20 |  | 45 | 27 |  |  |

JPY, Japanese Yen.

^a^ Chi-squared test for nominal variables and Wilcoxon-type test for trend for ordinal variables.

^b^ Categorized into four groups: low (<4 million JPY), lower-middle (≥4 million JPY to <6 million JPY), upper-middle (≥6 million JPY to <8 million JPY), and high (≥8 million JPY).

Table S2. Logistic regression analysis for factors associated with medical help-seeking among participants who had reported current fertility problems

|  |  | Univariable  (n=341) | | | | |  | Multivariable (complete case analysis)  (n=312) | | | | |  | Multivariable (multiple imputation)  (n=347) | | | | |
| --- | --- | --- | --- | --- | --- | --- | --- | --- | --- | --- | --- | --- | --- | --- | --- | --- | --- | --- |
|  |  | Odds ratio |  | 95%CI |  | p |  | Odds ratio |  | 95%CI |  | p |  | Odds ratio |  | 95%CI |  | p |
| Household income^a^ | |  |  |  |  |  |  |  |  |  |  |  |  |  |  |  |  |  |
|  | Low | ref |  |  |  |  |  | ref |  |  |  |  |  | ref |  |  |  |  |
|  | Lower-middle | 1.15 |  | 0.59-2.26 |  | 0.69 |  | 1.07 |  | 0.52-2.20 |  | 0.86 |  | 1.03 |  | 0.51-2.08 |  | 0.94 |
|  | Upper-middle | 1.64 |  | 0.80-3.36 |  | 0.18 |  | 1.68 |  | 0.76-3.72 |  | 0.20 |  | 1.47 |  | 0.68-3.16 |  | 0.32 |
|  | High | 1.64 |  | 0.83-3.23 |  | 0.15 |  | 1.61 |  | 0.72-3.59 |  | 0.25 |  | 1.58 |  | 0.72-3.46 |  | 0.26 |
| Wife's age, years | |  |  |  |  |  |  |  |  |  |  |  |  |  |  |  |  |  |
|  | ≤29 | ref |  |  |  |  |  | ref |  |  |  |  |  | ref |  |  |  |  |
|  | 30–34 | 1.73 |  | 0.81-3.70 |  | 0.16 |  | 1.50 |  | 0.67-3.38 |  | 0.33 |  | 1.78 |  | 0.80-3.96 |  | 0.16 |
|  | 35–39 | 1.68 |  | 0.81-3.47 |  | 0.16 |  | 1.49 |  | 0.66-3.39 |  | 0.34 |  | 1.55 |  | 0.69-3.48 |  | 0.29 |
|  | ≥40 | 2.18 |  | 1.02-4.67 |  | 0.05 |  | 1.72 |  | 0.72-4.08 |  | 0.22 |  | 1.93 |  | 0.82-4.53 |  | 0.13 |
| Husband's age, years | |  |  |  |  |  |  |  |  |  |  |  |  |  |  |  |  |  |
|  | ≤29 | ref |  |  |  |  |  | - |  | - |  | - |  | - |  | - |  | - |
|  | 30–34 | 1.05 |  | 0.45-2.47 |  | 0.90 |  | - |  | - |  | - |  | - |  | - |  | - |
|  | 35–39 | 1.53 |  | 0.68-3.46 |  | 0.31 |  | - |  | - |  | - |  | - |  | - |  | - |
|  | ≥40 | 1.56 |  | 0.71-3.43 |  | 0.27 |  | - |  | - |  | - |  | - |  | - |  | - |
| Length of the marriage, years | |  |  |  |  |  |  |  |  |  |  |  |  |  |  |  |  |  |
|  | ≤4 | ref |  |  |  |  |  | ref |  |  |  |  |  | ref |  |  |  |  |
|  | 5–9 | 1.69 |  | 1.04-2.72 |  | 0.03 |  | 1.87 |  | 1.07-3.26 |  | 0.03 |  | 1.67 |  | 0.98-2.86 |  | 0.06 |
|  | 10–14 | 1.44 |  | 0.75-2.75 |  | 0.27 |  | 1.62 |  | 0.76-3.45 |  | 0.22 |  | 1.62 |  | 0.77-3.39 |  | 0.20 |
|  | ≥15 | 1.20 |  | 0.46-3.12 |  | 0.72 |  | 1.70 |  | 0.53-5.43 |  | 0.37 |  | 1.15 |  | 0.40-3.32 |  | 0.80 |
| Wife's educational level | |  |  |  |  |  |  |  |  |  |  |  |  |  |  |  |  |  |
|  | Highschool education or less | ref |  |  |  |  |  | ref |  |  |  |  |  | ref |  |  |  |  |
|  | Vocational or junior college education | 1.44 |  | 0.85-2.43 |  | 0.17 |  | 1.14 |  | 0.64-2.03 |  | 0.66 |  | 1.21 |  | 0.70-2.11 |  | 0.49 |
|  | University education or higher | 1.43 |  | 0.81-2.52 |  | 0.21 |  | 1.21 |  | 0.64-2.29 |  | 0.55 |  | 1.26 |  | 0.68-2.32 |  | 0.46 |
| Husband's educational level | |  |  |  |  |  |  |  |  |  |  |  |  |  |  |  |  |  |
|  | Highschool education or less | ref |  |  |  |  |  | - |  | - |  | - |  | - |  | - |  | - |
|  | Vocational or junior college education | 1.75 |  | 0.96-3.21 |  | 0.07 |  | - |  | - |  | - |  | - |  | - |  | - |
|  | University education or higher | 1.61 |  | 1.00-2.60 |  | 0.05 |  | - |  | - |  | - |  | - |  | - |  | - |
| Wife's employment status | |  |  |  |  |  |  |  |  |  |  |  |  |  |  |  |  |  |
|  | Full-time worker | ref |  |  |  |  |  | ref |  |  |  |  |  | ref |  |  |  |  |
|  | Part-time worker | 1.38 |  | 0.81-2.35 |  | 0.23 |  | 1.65 |  | 0.89-3.04 |  | 0.11 |  | 1.82 |  | 1.00-3.30 |  | 0.05 |
|  | Self-employed | 0.74 |  | 0.22-2.48 |  | 0.63 |  | 0.91 |  | 0.24-3.39 |  | 0.89 |  | 0.80 |  | 0.23-2.86 |  | 0.73 |
|  | Unemployed | 0.80 |  | 0.47-1.36 |  | 0.40 |  | 1.10 |  | 0.58-2.10 |  | 0.77 |  | 0.93 |  | 0.50-1.73 |  | 0.82 |
| Husband's employment status | |  |  |  |  |  |  |  |  |  |  |  |  |  |  |  |  |  |
|  | Full-time worker | ref |  |  |  |  |  | - |  | - |  | - |  | - |  | - |  | - |
|  | Part-time worker | 1.02 |  | 0.48-2.15 |  | 0.97 |  | - |  | - |  | - |  | - |  | - |  | - |
|  | Self-employed | 0.50 |  | 0.24-1.04 |  | 0.07 |  | - |  | - |  | - |  | - |  | - |  | - |
|  | Unemployed | 0.72 |  | 0.16-3.26 |  | 0.67 |  | - |  | - |  | - |  | - |  | - |  | - |
| Number of existing children | |  |  |  |  |  |  |  |  |  |  |  |  |  |  |  |  |  |
|  | 0 | ref |  |  |  |  |  | ref |  |  |  |  |  | ref |  |  |  |  |
|  | 1 | 0.96 |  | 0.60-1.54 |  | 0.86 |  | 0.83 |  | 0.49-1.41 |  | 0.49 |  | 0.84 |  | 0.50-1.40 |  | 0.49 |
|  | 2 | 0.19 |  | 0.04-0.88 |  | 0.03 |  | 0.11 |  | 0.02-0.57 |  | 0.01 |  | 0.11 |  | 0.02-0.59 |  | 0.01 |
|  | ≥3 | NA |  |  |  |  |  | NA |  |  |  |  |  | NA |  |  |  |  |
| Living with their parents | |  |  |  |  |  |  |  |  |  |  |  |  |  |  |  |  |  |
|  | Yes | 1.26 |  | 0.77-2.06 |  | 0.36 |  | - |  | - |  | - |  | - |  | - |  | - |
|  | No | ref |  |  |  |  |  | - |  | - |  | - |  | - |  | - |  | - |
| Residential region | |  |  |  |  |  |  |  |  |  |  |  |  |  |  |  |  |  |
|  | Hokkaido | ref |  |  |  |  |  | - |  | - |  | - |  | - |  | - |  | - |
|  | Tohoku | 0.50 |  | 0.13-1.88 |  | 0.31 |  | - |  | - |  | - |  | - |  | - |  | - |
|  | Kanto | 0.72 |  | 0.24-2.22 |  | 0.57 |  | - |  | - |  | - |  | - |  | - |  | - |
|  | Chubu | 0.65 |  | 0.21-2.07 |  | 0.47 |  | - |  | - |  | - |  | - |  | - |  | - |
|  | Kinki | 0.75 |  | 0.23-2.44 |  | 0.63 |  | - |  | - |  | - |  | - |  | - |  | - |
|  | Chugoku/Shikoku | 0.84 |  | 0.24-2.91 |  | 0.78 |  | - |  | - |  | - |  | - |  | - |  | - |
|  | Kyushu/Okinawa | 1.13 |  | 0.30-4.24 |  | 0.86 |  | - |  | - |  | - |  | - |  | - |  | - |
| Population size and density | |  |  |  |  |  |  |  |  |  |  |  |  |  |  |  |  |  |
|  | Non-densely inhabited district | ref |  |  |  |  |  | - |  | - |  | - |  | - |  | - |  | - |
|  | <200,000 inhabitants | 1.28 |  | 0.70-2.34 |  | 0.42 |  | - |  | - |  | - |  | - |  | - |  | - |
|  | 200,000 to 1,000,000 inhabitants | 1.10 |  | 0.60-2.02 |  | 0.76 |  | - |  | - |  | - |  | - |  | - |  | - |
|  | ≥1,000,000 inhabitants | 1.66 |  | 0.89-3.12 |  | 0.11 |  | - |  | - |  | - |  | - |  | - |  | - |

CI, confidence interval; JPY, Japanese Yen; NA, not assessed; ref, reference.

^a^ Categorized into four groups: low (<4 million JPY), lower-middle (≥4 million JPY to <6 million JPY), upper-middle (≥6 million JPY to <8 million JPY), and high (≥8 million JPY).
